# Determinants of Caregivers’ Ability to Accurately Detect Acute Malnutrition Using Color-Coded Mid-Upper Arm Circumference Tape and Pitting Edema

**DOI:** 10.1016/j.cdnut.2025.107477

**Published:** 2025-05-28

**Authors:** Benson C Singano, Collina A Tchongwe, Numeri C Geresomo, Tinna Ng’ong’ola-Manani, Aaron T Chikakuda, Alfred Ngwira, Stanley Mwase, Elsie Mawala, Benson Kazembe, Emma Budalla, Alexander A Kalimbira

**Affiliations:** 1 Department of Human Nutrition and Health, Lilongwe University of Agriculture and Natural Resources, Lilongwe, Malawi; 2 Screening and Referral by Caregivers Project, Lilongwe University of Agriculture and Natural Resources, Lilongwe, Malawi; 3 Department of Food Science and Technology, Lilongwe University of Agriculture and Natural Resources, Lilongwe, Malawi; 4 Department of Basic Sciences, Lilongwe University of Agriculture and Natural Resources, Lilongwe, Malawi; 5 Nutrition section, United Nations Children's Fund, Lilongwe, Malawi

**Keywords:** caregivers, children, acute malnutrition, nutritional screening, MUAC tape

## Abstract

**Background:**

In Malawi, inadequate community health workers are delaying admission of acute malnutrition cases into therapeutic programs. Training caregivers to screen their children for early identification of acute malnutrition has been shown to improve early admissions, prevent serious complications, and save lives. However, there are knowledge gaps regarding the determinants of caregivers to accurately screen for acute malnutrition.

**Objectives:**

To identify determinants of accuracy in detecting acute malnutrition using color-coded mid-upper arm circumference (MUAC) tape and pitting edema among caregivers.

**Methods:**

This was a cross-sectional study taken as a baseline for a larger non-random pragmatic interventional study. Using a cascade model, community health workers (health surveillance assistants, *n* = 148) from 2 southern districts of Nsanje and Phalombe in Malawi, trained 12,057 caregivers of children aged 6 to 54 months in nutritional screening using color-coded MUAC tapes and pitting edema. Pretested structured questionnaires were used in face-to-face interviews with the caregivers to collect data on their age, marital status, education, district, occupation, and age of the child, which were predictor variables. Caregivers’ accuracy was the response variable. Binary logistic regression was used to assess the effect of each predictor variable on accuracy of caregivers.

**Results:**

Compared to caregivers who were farmers (86.2%), those who were formally employed (1.8%) were nearly 70% less likely to accurately determine acute malnutrition [adjusted odds ratio (AOR): 0.30; 95% confidence interval (CI): 0.12, 0.75] than farmers. Caregivers from Phalombe district were nearly 5 times more likely to be accurate than caregivers from Nsanje district (AOR: 4.93; 95% CI: 3.31, 7.35). Caregivers were twice and thrice more likely to accurately screen children aged 31–42 mo (AOR: 2.44; 95% CI: 1.43, 4.17) and 43–54 mo (AOR: 2.83; 95% CI: 1.45, 5.54), respectively, than children aged 6–11 mo.

**Conclusions:**

The outcome of training caregivers to use color-coded MUAC tapes and pitting edema to screen for acute malnutrition is likely to be sensitive to several factors including residence, occupation of the caregivers, and age of the children. More data and further studies are required to validate the present results.

## Introduction

Children with severe acute malnutrition (SAM) are 8 times more likely to die than well-nourished children [1]. The Community-Based Management of Acute Malnutrition (CMAM) program has assisted in the management of acute malnutrition in an attempt to reduce child mortality. Through the CMAM program, community health workers (CHWs) use mid-upper arm circumference (MUAC) tapes to identify cases of acute malnutrition for referral and treatment [2,3]. The MUAC tape measures the circumference of the midpoint of the upper arm between the shoulder bone and the elbow, and identifies reductions in muscle mass and subcutaneous fat [4].

The CHWs are trusted to screen for acute malnutrition with fewer and minimal errors under field conditions [5]. Similarly, caregivers have demonstrated capacity to screen for acute malnutrition using color-coded MUAC tapes and pitting edema with minimal training [6], resulting in reduced hospitalization due to early detection [2].

Evidence suggests that there is late identification of cases of acute malnutrition even in areas with functional CMAM programs [7]. One of the contributing factors is that there are fewer CHWs than required, and are overwhelmed with heavy workloads [7,8]. Strengthening caregivers’ capacity to screen and refer for appropriate management is a promising alternative to achieving early identification of cases of acute malnutrition [6], and the caregivers can be trusted with the responsibility to monitor the nutritional status of their children [2]. However, there remain knowledge gaps regarding the determinants of caregivers to accurately screen for malnutrition. Therefore, this study aimed to identify determinants of caregivers to accurately detect acute malnutrition using color-coded MUAC tapes and pitting edema.

## Methods

### Study design

This was a cross-sectional study taken as baseline for larger non-random pragmatic interventional study which lasted 6 mo. The study took place in 2 southern districts of Nsanje and Phalombe in Malawi. Two traditional authorities (TAs) of Ndamera and Tengani in Nsanje district, and Jenala and Mkhumba in Phalombe district were purposively sampled based on the priority of the Screening and Referral by Caregivers (SCRECA) project that was being implemented by the Lilongwe University of Agriculture and Natural Resources (LUANAR) with funding from the United Nations Children’s Fund (UNICEF). Participants were caregivers who included mothers, fathers, guardians, and females of childbearing age who had children aged 6–54 mo in their households. The 6-mo study used a closed cohort to ensure that the recruited children remained eligible as under-fives to the end of the study.

### Sample population

The study recruited all caregivers of children aged 6–54 mo residing in Phalombe (TAs Mkhumba and Jenala) and Nsanje (TAs Tengani and Ndamera) districts in southern Malawi, who provided consent and were available to participate in the study. The study areas were chosen because they were districts with livelihood with highest caseloads of acute malnutrition (6.6%) [9].

### Training of trainers and caregivers

The study adopted a cascade model of trainings from district to community levels and reaching caregivers. In Stage 1, the study investigators from LUANAR trained District Nutrition Coordinating Committee (DNCC) members in Phalombe and Nsanje districts. The DNCC is a technical multisectoral district level committee comprising various nutrition stakeholders and is responsible for coordinating implementation of nutrition policies and programs. In each DNCC, 8 members with prior training in nutrition were trained as trainers of trainers. In Phalombe district, the training took 2 d: on Day 1, the participants covered theory work on procedures for using MUAC tapes and how to assess pitting edema (Supplemental Figures 1–12). On Day 2, participants were taken to a nearby community to practice screening children using MUAC tapes and pitting edema. In Nsanje district, theoretical learning and practice of screening children using MUAC tapes and pitting edema were done in 1 d.

In Stage 2, the trained DNCC members cascaded the training to health-sector CHWs known as health surveillance assistants (HSAs) and community volunteers known as care group promoters (CGPs) under the supervision of the investigators. In Phalombe district, the training took 2 d: on Day 1, the participants covered theory on procedures for using MUAC tapes and how to assess pitting edema. On Day 2, participants were taken to a nearby community to practice screening children using MUAC tapes and pitting edema. In Nsanje district, theoretical learning and practice of screening children using MUAC tapes and pitting edema were done in 1 d.

In Stage 3, a team comprising HSAs and CGPs trained caregivers in their communities under the supervision of the DNCC and the investigators. CGPs mobilized caregivers into groups of 30 per training session. The theory and demonstrations took utmost 50 min, whereas practice and data collection took ∼90 min. First, the training involved a discussion on infant and young child feeding practices, acute malnutrition, use of MUAC tape, and edema screening (Supplemental Tables 1–4). Second, the HSAs and CGPs demonstrated use of a MUAC tape and assessment of edema while the caregivers were observing (Supplemental Figures 13–18). Third, the caregivers practiced under the supervision of the HSAs and the CGPs. Finally, the caregivers reported their screening results (MUAC color codes and categories of pitting edema) which HSAs recorded in pre-designed forms (Supplemental Figure 13). In this stage, roles of the HSAs included facilitating training and recording data generated by the caregivers, whereas the CGPs assisted caregivers in screening.

Development of training protocols went through a process, and the process included the following: 2 primary researchers identified areas of interest based on the research questions, which included nutritional screening of under-five children by caregivers using color-coded MUAC tapes and bilateral pitting edema; available treatments for acute malnutrition within the CMAM program; possible causes of malnutrition; and referral systems (BCS,CAT). The 2 researchers divided writing tasks to address these areas of interest, exchanging their work for review and content validation (BCS,CAT). Subsequently, the researchers consolidated their insights and compiled the initial drafts of training protocols (BCS,CAT). These drafts were sent to 4 senior researchers for review and content validation (NCG,TN-M,ATC,AAK). The senior researchers provided guidance on the validity of the training protocols in relation to the overall objectives of the study. The content was adapted from original articles, ALIMA training manuals, and the CMAM training manual developed for MUAC training. The training protocols were tested on nutrition implementers at district level, and improvements were made to suit how nutrition programs are implemented at community level.

### Data analysis

Cross tabulations were used to describe characteristics of study participants, and Pearson Chi-square test was used to determine significant differences among categories of the characteristics. Severe acute malnutrition, moderate acute malnutrition, and global acute malnutrition (GAM) were determined using MUAC <11.5 cm (red color code) and presence of bilateral pitting edema, MUAC between 11.5 cm and 12.4 cm (yellow color code), and MUAC <12.4 cm (red and yellow color code), respectively (Supplementary Figure 13) [10]. Binary logistic regression was used to assess the effect of explanatory variables on caregivers’ accuracy in identifying children with acute malnutrition, as compared to assessments done by CHWs [11]. Caregivers were considered accurate (1 = “accurate”; 0 = “not accurate”) when their measurements matched those of CHWs. Binary logistic regression analysis was chosen because the response variable was binary (accurate and not accurate). A *P* value of <5% was used as a test of significance. Explanatory variables included educational level, ages of caregivers and children, occupation of caregivers, and district of residence. The prevalence of missing data across variables was as follows: education (*n* = 15; 0.1%), district of residence (*n* = 0; 0%), marital status (*n* = 9; 0.1%), occupation (*n* = 4; 0.1%), caregiver age categories (*n* = 49; 0.4%), child age categories (*n* = 280; 2.3%), and accuracy (*n* = 73; 0.6%). Listwise deletion method was automatically applied during the analysis using IBM SPSS Statistics version 20.0, which enabled each analysis to maximize the use of available data without entirely excluding cases with missing values in some variables These variables were assessed based on their relevance to study and significance of association with the response variable. The Omnibus Test of model coefficient was used to determine the significant contribution of explanatory variables that significantly contributed to the binary logistic regression model, with significance determined by a *P* value of <0.05 (10). The *P* value of Omnibus Tests of Model Coefficient in this study was <0.01. This *P* value indicated that the model with the predictors is significantly better than the baseline model without predictors [11]. The Hosmer and Lemmeshow Test was used to test goodness-of-fit of the model and the goodness-of-fit of the model to assess how well the model predicts outcomes, and it was determined by the *P* value of >0.05 [11]. The *P* value of Hosmers-Lemeshow test in this study was *P* = 0.788 which was not significant at 5%, implying that the model fits the data well, and that there was no significant difference between observed and expected frequencies [11]. Explanatory variables were also tested against the response variable to ascertain significant association between the explanatory and response variable using Pearson Chi-square test, with a *P* value < 0.05 [11]. Multicollinearity among explanatory variables was tested by examining associations using Pearson Chi-square test (*P* < 0.05). Explanatory variables with significant associations were selected [12]. Chi-square tests of associations were used to test the significance of association among variables, as all had >5 counts in cross tabulations [12]. The Enter Method was used to include explanatory variables in the binary logistic regression model, and significance of each variable was determined (*P* < 0.05). Data analysis was performed using IBM SPSS Statistics (Version 20.0). The binary response of accuracy in screening nutritional status among the caregivers was determined using the binary logistic regression model below:

$$\pi_{i}=P\left(Y=1|\;x_{1}\right)=\frac{e^{\beta_{0}+\beta_{1_{X}1}+\beta_{2_{X}2}+\ldots +\beta_{p_{X}p}}}{1+e^{\beta_{0}+\beta_{1_{X}1}+\beta_{2_{X}2}+\ldots +\beta_{p_{X}p}}}$$

Let $\pi_{i}$ be a response variable representing odds of the *i*^*th*^ caregiver accurately screening nutritional status of a child for a given $X_{i.}$ P represents probability. Y = 1 represents the accurate screening of nutritional status by a caregiver. $X_{i}$ ($X_{1,}X_{2},\ldots X_{p}$) is an explanatory variable of the *i*^*th*^ caregiver. $P\left(x_{1}\right)=$ the probability of the *i*^*th*^ caregiver accurately screening nutritional status of a child for a given $X_{i.}$ β (β_1,_ β_2,…_ β_p_) are parameters of the model and β_0_ is an intercept, but altogether are regression coefficients [13].

### Ethical consideration

Ethical approval was granted by the National Health Sciences Research Committee (NHSCR) in Malawi with approval number 21/08/2757. Permission to conduct the study was granted by district commissioners, and local leaders in the respective districts, and additionally in Nsanje, the district health management team. Initially, the “Family MUAC” project, as it was called, was not aimed at generating publishable evidence but to provide a “Do-it-yourself” type of opportunity to caregivers to assess their children’s nutritional status. Initially, this did not require ethical clearance. However, given the significance of the intervention and the scientific rigor that was applied, the research team decided that the ensuing results could be useful to the scientific community, hence we embarked on post facto ethical approval. We are well aware that a priori ethical approval is the recommended practice. With that background, we recruited the first participants on October 3, 2020, while ethical approval was granted on November 21, 2021. At the on-set of the project, we were granted permission by Phalombe and Nsanje district councils during entry meetings held on September 28 in Phalombe, and September 29, 2020, in Nsanje district. All caregivers who participated in the study gave informed consent. For caregivers who were below the age of 18 y, we obtained consent directly from them as mothers of their children. In total, there were 139 (1.2%) caregivers participants who were under the age of 18 y. Phalombe district practices matrilineal systems of marriage, whereas Nsanje practices patrilineal system. This means that the adolescent mother in Phalombe would likely be living proximal to her parents (grandparents of her child), whereas in Nsanje, the caregiver would likely be distal to her parents.

## Results

### Characteristics of caregivers

In total, there were 12,057 caregivers (*n* = 4182 in Nsanje; *n* = 7875 in Phalombe) who participated in the study. The participants were mostly aged 25–39 y; had largely attained primary education (72.6%); mostly married (87.3%); and were involved in farming (86.2%) as their main source of livelihood (Table 1).

**TABLE 1:** Characteristics of caregivers disaggregated by district of residence.

Characteristics	Nsanje *n* (%)	Phalombe *n* (%)	Total *n* (%)	Chi-square	*P* value
Age of caregivers (y)				23.645	<0.01^1^
14–17	54 (1.3)	85 (1.1)	139 (1.2)		
18–24	1755 (42.5)	3433 (43.6)	5188 (43.2)		
25–39	2015 (48.8)	3938 (50.0)	5953 (49.6)		
40–77	309 (7.5)	419 (5.3)	728 (6.1)		
Total	4133 (100)	7875 (100)	12,008 (100)		
Education level				1287.339	<0.01^1^
No education	1147 (27.5)	405 (5.1)	1552 (12.9)		
Primary education	2388 (57.3)	6357 (80.7)	8745 (72.6)		
Secondary education	592 (14.2)	1089 (13.8)	1681 (14)		
Tertiary education	40 (1.0)	24 (0.3)	64 (0.5)		
Total	4167 (100)	7875 (100)	12,042 (100)		
Residence within the district					
TA Ndamera	1771 (47.3)	-	1771 (14.7%)		
TA Jenala	-	4281 (54.4)	4281 (35.5)		
TA Mkhumba	-	3594 (45.6%)	3594 (29.8)		
TA Tengani	2411 (57.7)	-	2411 (20.0)		
Total	4182 (100)	7875 (100)	12,057 (100)		
Marital status				119.692	<0.01^1^
Married	3608 (86.4)	6910 (87.8)	10,518 (87.3)		
Unmarried	567 (13.6)	963 (12.2)	1530 (12.7)		
Total	4175 (100)	7873 (100)	12,048 (100)		
Occupation				136.377	<0.01^1^
Business	516 (12.4)	673 (8.5)	1189 (9.9)		
Employed	48 (1.1)	165 (2.1)	213 (1.8)		
Farmer	3458 (82.8)	6933 (88.0)	10,391 (86.2)		
Others	156 (3.7)	104 (1.3)	260 (2.2)		
Total	4178 (100)	7875 (100)	12,053 (100)		

1 Significant at 5%.

### Characteristics of children

In total, there were 16,956 children (*n* = 5622 in Nsanje; *n* = 11,334 in Phalombe) who were assessed by their caregivers. More than half of the children (53.6%) were in the combined ages of 12–23 mo (27.8%) and 24–35 mo (25.8%). Overall, less than half of the children (48.8%) were boys, although there were proportionately more female children in Phalombe (52.5%) than Nsanje. Prevalence of severe, moderate, and GAM were the same in the 2 districts (Table 2).

**TABLE 2:** Characteristics of children disaggregated by district of residence.

Characteristics	Nsanje *n* (%)	Phalombe *n* (%)	Total *n* (%)	Chi-square	*P* value
Age (mo)				125.923	<0.01^1^
6–11	787 (19.0)	1206 (15.8)	1993 (16.9)		
12–23	1294 (31.2)	1983 (26.0)	3277 (27.8)		
24–35	1071 (25.8)	1968 (25.8)	3039 (25.8)		
36–47	646 (15.6)	1406 (18.4)	2052 (17.4)		
48–59	347 (8.4)	1069 (14.0)	1416 (12.0)		
Total	4145 (100)	7632 (100)	11,777(100)		
Sex (*n*)				32.066	<0.001^1^
Male	2918 (51.9)	5389 (47.5)	8307 (48.8)		
Female	2704 (48.1)	5945 (52.5)	8649 (51.2)		
Total	5622 (100)	11,334 (100)	16,956 (100)		
Prevalence of acute malnutrition				0.015	1.000
SAM^2^	10 (0.2)	18 (0.2)	28 (0.2)		
MAM^3^	72 (1.7)	136 (1.7)	208 (1.7)		
GAM^4^	82 (1.9)	154 (1.9)	236 (1.9)		

Accuracy was defined as the ability of a caregiver to correctly classify a child as malnourished (SAM or MAM) or not malnourished (normal MUAC).

Abbreviations: GAM, global acute malnutrition; MAM, moderate acute malnutrition; MUAC, mid-upper arm circumference; SAM, severe acute malnutrition.

1 Significant difference in the prevalence at 5% significance level.

2 SAM (red color code on MUAC referring to MUAC <11.5 cm, and/or presence of bilateral pitting edema).

3 MAM (yellow color code on MUAC referring to MUAC within the range of 11.5–12.4 cm).

4 GAM (both red and yellow codes on MUAC referring to MUAC <12.4 cm and/or presence of bilateral pitting edema).

### Determinants of caregivers to accurately detect acute malnutrition

Nearly all measurements taken by caregivers (98.7%) were in agreement with those of HSAs, more so in Phalombe district (99.5%) than in Nsanje district (97.2%). Table 3 shows the determinants of accuracy of caregivers to screen for acute malnutrition among their children. The model was adjusted with the following factors: education level, occupation, and age of caregivers; and age of children and their district of residence. Goodness-of-fit of the model was evaluated by Hosmers-Lemeshow test (*P* = 0.788), suggesting that the model was fit. Compared with caregivers who were farmers (86.2%), those who were formally employed (1.8%) were nearly 70% less likely to accurately determine acute malnutrition [adjusted odds ratio (AOR): 0.30; 95% CI: 0.12, 0.75]. Caregivers from Phalombe district were nearly 5 times more likely to be accurate than caregivers from Nsanje district (AOR: 4.93; 95% CI: 3.31, 7.35). Caregivers were twice and thrice more likely to accurately screen children aged 31–42 mo (AOR: 2.44; 95% CI: 1.43, 4.17) and 43–54 mo (AOR: 2.83; 95% CI: 1.45, 5.54), respectively, than children aged 6–11 mo.

**TABLE 3:** Factors affecting accuracy of caregivers to screen for nutrition status.

Factors	AOR (95% CI)^1^	*P* value
District of residence		
Nsanje	1.00	
Phalombe	4.926 (3.305, 7.347)	<0.001^2^
Occupation of caregiver		
Farmers	1.000	
Employed	0.302 (0.122, 0.749)	0.01^2^
Business	1.184 (0.712, 1.968)	0.51
Others	0.515 (0.226, 1.176)	0.11
Age of children (mo)		
6–11	1.00	
12–23	1.102 (0.720, 1.688)	0.66
24–35	1.700 (1.050, 2.754)	<0.01^2^
36–47	3.154 (1.621, 6.134)	<0.01^2^
48–59	2.009 (1.028, 3.926)	0.04^2^
Age of caregivers (y)		
<18	1.000	
19–25	0.649 (0.088, 4.778)	0.672
26–39	0.398 (0.054, 2.909)	0.364
40>	0.284 (0.037, 2.200)	0.228
Education level		
No education	1.000	
Primary education	1.474 (0.997, 2.179)	0.070
Secondary education	1.854 (0.995, 3.294)	0.052
Tertiary education	0.914 (0.216, 3.951)	0.856

Abbreviations: AOR, adjusted odds ratio; CI, confidence interval.

1 AOR and 95% CIs for factors associated with accuracy in screening for acute malnutrition. The model was adjusted with the following explanatory variables; marital status, age of caregivers, and educational level. *P* value for Omnibus Tests of Model Coefficient was significant at 5% (*P* < 0.01). Hosmer and Lemmeshow had a *P* value of 0.488. District of residence, occupation of caregiver, age of children, and educational level had a *P* value of <5% in the test of determining significance of each explanatory variable to the model.

2 Significant different from a reference group at 5% significance level.

## Discussion

In this study, we aimed to identify determinants of caregivers to accurately detect acute malnutrition using color-coded MUAC tape and pitting edema among children of caregivers from 2 southern districts of Nsanje and Phalombe in Malawi. Almost all caregivers’ measurements were in agreement with those of HSAs in both districts. In the 2 study sites Phalombe and Nsanje districts, the sensitivities for SAM were 94.4% and 27.2%, whereas specificities were 99.9% and 100%, respectively. Similarly, the sensitivities for GAM were 85.6% and 51.8%, whereas specificities of 99.98% and 98.7%, respectively. In Niger, a study of 103 children and their caregivers showed that the caregivers were able to screen their children for acute malnutrition, with sensitivity (>90%) and specificity (>80%) for classifying GAM [6]. The MUAC tape has been shown to be easy to learn and use even by people with low education/socioeconomic [6]. In the present study, the participants were resident in remote areas, had low education, and were subsistent farmers. Such a socio-demographic profile gives confidence that the color-coded MUAC tape can be effectively used to enhance screen of acute malnutrition by caregivers. The proficiency in using the MUAC tape was accomplished through a cascaded training model using existing nutrition coordinating structures at district and community levels, CHWs, and community volunteers.

Three factors (district of residence, occupation of caregivers, and age of children) were identified as significant determinants of caregivers’ accuracy in identifying cases of acute malnutrition. Caregivers from Phalombe were more likely to identify cases of acute malnutrition than those from Nsanje district. We believe that district of residence was a proxy of other more directly important factors such as educational attainment and literacy. The study results showed that there were 5.4 times more of uneducated caregivers in Nsanje than the Phalombe district (Table 1). The 2015/16 Malawi Demographic and Health Survey showed that 24.8% and 10.8% of females aged 15–49 y from Nsanje and Phalombe districts, respectively, had no formal education [9]. Further, the 2018 Malawi Population and Housing Census showed that 56% and 65% of people aged 5 y and older in Nsanje and Phalombe districts, respectively, were literate [14]. This implies that caregivers form Nsanje were more disadvantaged than those from Phalombe. Although education was not a significant determinant, uneducated caregivers are less likely to comprehend the theory of MUAC and malnutrition and, therefore, have difficulties in mastering the skill in screening for acute malnutrition using the MUAC tape and pitting edema. Elsewhere studies have shown that mothers who lacked literacy skills were less likely to achieve recommended complementary feeding indicators [15].

The results that caregivers who were formally employed were less likely to accurately identify their acutely malnourished children was surprising. It is generally expected that formally employed females would have a little more education than others, and they would comprehend new knowledge and master skills better and faster than others. In the present study, HSAs who conducted the trainings observed that caregivers who were formally employed had a better socioeconomic status and had a perception that their children could not be malnourished. As such, they were reporting late for the MUAC trainings, and were less attentive during the training sessions. This could be the reason for their poor performance. Evidence indicates that improved maternal education is associated with better child-care practices related to health and nutrition, reduced odds of stunting, and better ability to access and benefit from interventions [16]. However, there is limited information on the association of formal employment and socioeconomic status among caregivers in rural communities, and association of formal employment and participation of caregivers in programs that aim to end acute malnutrition in rural communities. Overall, the proportion of formally employed caregivers was only 1.8%, whereas that of farmers was 86.2% (Table 1). Statistically, the few numbers of formally employed females would be insufficient to have positive effect on the outcome [17].

The capacity of caregivers to accurately identify cases of acute malnutrition increased with increasing age of children. Caregivers who had children aged 2 y and older were more likely to identify acute malnutrition than caregivers with children below the age of 2 y. During training, HSAs observed that younger children were easily agitated during screening resulting in into jerky movements which affected accuracy of measurements. Gupta and colleagues reported that measuring height was more difficult among children below the age of 2 y than older ones because the younger children were less cooperative [18]. This supports our findings that children below the age of 2 y are more difficult to measure. Therefore, caregivers of younger children would need to be more careful and patient when handling their children. Furthermore, such caregivers may benefit more from frequent supportive supervision by HSAs or CGPs.

Our study had several limitations. First, the study had limited number of social, demographic and economic factors, and other characteristics of the children, caregivers, and their households. As such, there was limited ability to identify more significant determinants of accurate assessment. Second, the number of formally employed caregivers was very small—1.8% of the sample of caregivers in the 2 districts. This may have masked the effect of formal employment on accuracy of caregivers to assess acute wasting. Third, our results are based on a lot of supervision of HSAs and CGPs by DNCC and the research team, as they trained the caregivers. The study should therefore be considered efficacious and not effectiveness in nature. Finally, the data reflect practices of caregivers and HSAs/CGPs over a period of 6 mo. A longer-term implementation study may generate varying results.

In this study, we have demonstrated that trained caregivers of children below the age of 5 y can accurately use color-coded MUAC tapes and pitting edema to screen for acute malnutrition among their children. Although the results of our study are promising, the use of color-coded MUAC tapes by caregivers in a low-income setting required validation in varied contexts. Future researchers may consider longitudinal study designs with the aim of assessing whether caregiver accuracy in malnutrition detection changes over time with repeated training, experience, and continued presence or absence of acute malnutrition in their children. The study would follow the same caregivers over months or years to evaluate retention of skills and identify factors that influence sustained accuracy or lack of it. Despite the aforementioned limitations, the use of color-coded MUAC tapes and pitting edema may be rolled out in other districts in Malawi using the cascade model.

## Author contributions

The authors’ responsibilities are as follows – AAK, SM, BK, and EM conceptualized the study; BCS, CAT, NCG, TN-M, ATC, AN, SM, EM, BK, EB, and AAK designed study; BCS, CAT, EB, and AAK conducted the research; BCS, CAT, and AN performed statistical analyses; BCS, CAT, NCG, TN-M, ATC, AN, SM, EM, BK, EB, and AAK had primary responsibility for the final content, thoroughly reviewed the manuscript, and approved the final version.

## Data availability

Data described in the article will be made available upon request, pending application and approval.

## Funding

UNICEF Malawi office provided funding for the research. The funding bodies had no involvement in the research design, data gathering and analysis, publication decisions, or manuscript preparation. The authors bear sole responsibility for the content, which may not necessarily reflect UNICEF’s official stance.

## Conflict of interest

AAK reports financial support was provided by UNICEF. EM reports a relationship with UNICEF that includes: employment. SM reports a relationship with UNICEF that includes: employment. BK reports a relationship with UNICEF that includes: employment. UNICEF Malawi office provided funding for the research. The funding bodies had no involvement in the research design, data gathering and analysis, publication decisions, or manuscript preparation. The authors bear sole responsibility for the content, which may not necessarily reflect UNICEF’s official stance. If there are other authors, they declare that they have no known competing financial interests or personal relationships that could have appeared to influence the work reported in this article.
