# Supplementary material for: Determinants of Caregivers’ Ability to Accurately Detect Acute Malnutrition Using Color-Coded Mid-Upper Arm Circumference Tape and Pitting Edema
Source: Curr Dev Nutr. 2025 May 28;9(7):107477. doi: 10.1016/j.cdnut.2025.107477 (PMC12221631; doi:10.1016/j.cdnut.2025.107477)
Supplement: Multimedia component 1 [file mmc1.docx]

Screening and Referral by Caregivers (SCRECA) Project

**Manual for Training of Trainers**

*Implemented by*

Lilongwe University of Agriculture and Natural Resources *in Partnership with* the Government of Malawi, Hunger Project and Unicef

|  |  |  |  |
| --- | --- | --- | --- |

TABLE OF CONTENTS

[DETERMINANTS OF CAREGIVERS’ ABILITY TO ACCURATELY DETECT ACUTE MALNUTRITION USING COLOR-CODED MUAC TAPE AND PITTING OEDEMA i](#_Toc195808929)

[CHAPTER ONE: SCRECA PROJECT 1](#_Toc195808930)

[What is SCRECA? 1](#_Toc195808931)

[What is the aim of SCRECA? 1](#_Toc195808932)

[Who will work with SCRECA project in the communities? 1](#_Toc195808933)

[CHAPTER TWO: OVERVIEW OF MALNUTRITION IN MALAWI 3](#_Toc195808934)

[What is malnutrition? 3](#_Toc195808935)

[Prevalence of Malnutrition in Malawi 3](#_Toc195808936)

[What causes malnutrition? 3](#_Toc195808937)

[What are the types of undernutrition? 4](#_Toc195808938)

[CHAPTER THREE: ACUTE MALNUTRITION 5](#_Toc195808939)

[What is acute malnutrition? 5](#_Toc195808940)

[What are the clinical forms of acute malnutrition? 5](#_Toc195808941)

[Effects of wasting 5](#_Toc195808942)

[Effects of nutritional edema (kwashiorkor) 6](#_Toc195808943)

[Categories of acute malnutrition 7](#_Toc195808944)

[Screening for acute malnutrition 7](#_Toc195808945)

[What is MUAC? 7](#_Toc195808946)

[What is the principle behind MUAC? 7](#_Toc195808947)

[What do the colour codes on a MUAC tape mean? 8](#_Toc195808948)

[8](#_Toc195808949)

[How can one interpret the colour codes on the MUAC tape? 9](#_Toc195808950)

[Advantages of using MUAC 10](#_Toc195808951)

[How does one take a MUAC measurement? 10](#_Toc195808952)

[When should caregivers check MUAC and oedema in their children? 12](#_Toc195808953)

[How can caregivers ensure safety of the MUAC tape? 12](#_Toc195808954)

[CHAPTER FOUR: COMMUNITY-BASED MANAGEMENT OF ACUTE MALNUTRITION (CMAM) 15](#_Toc195808955)

[What are the components of CMAM? 15](#_Toc195808956)

[How does SCRECA fit in CMAM program? 15](#_Toc195808957)

[CHAPTER FIVE: PROJECT DESIGN 17](#_Toc195808958)

[Study design 17](#_Toc195808959)

[Handling caregivers with false positives 17](#_Toc195808960)

[REFERENCES 19](#_Toc195808961)

# **CHAPTER** **ONE: SCRECA PROJECT**

## **What is SCRECA?**

SCRECA is an acronym which stands for Screening and Referral by Caregivers. It is a project funded by UNICEF and implemented by Lilongwe University of Agriculture and Natural Resources (LUANAR) in collaboration with Hunger project, Ministry of Health and Department of Nutrition and HIV/AIDS.

## **What is the aim of SCRECA?**

SCRECA aims at strengthening the efforts of CMAM in light of controlling the prevalence of acute malnutrition by strengthening active case finding to ensure early identification and referral for treatment of children with acute malnutrition in the areas of humanitarian shocks. SCRECA is targeting caregivers who will be trained in assessing nutritional status of their children using MUAC tapes and bilateral pitting oedema. It is believed that mothers or fathers are in the best position to detect the early signs of malnutrition since they are custodians of their children. Moreover, research in some countries shows that they can reliably measure MUAC if well trained (Blackwell, et al. 2015)

## **Who will work with SCRECA project in the communities?**

1. District Nutrition Coordinating Committee (DNCC) members
2. Health Surveillance Assistants (HSA)/care group promoter

As custodians of communities in delivering health services, HSAs will be focal people to do the following duties:

- Training caregivers in assessing nutritional status of 6-59 months children using MUAC tapes.
- Recording number of households that receive MUAC tapes
- Confirming cases of Moderate Acute Malnutrition (MAM) and Severe Acute Malnutrition (SAM) referred by caregivers and referring them for appropriate care.
- Referring confirmed cases of acute malnutrition
- Compiling monthly records on referred cases, true positives and false positives
- Compiling monthly reports on monitoring of usage of MUAC tapes by caregivers
- Commending caregivers who are assessing their children on regular basis

1. Caregivers

These are the target group for the project. With care group promoters and HSAs, they will be expected to do the following:

- Assess nutritional status of their children on regular basis
- Report cases of MAM and SAM to care group promoters and HSAs
- Report missing or destroyed MUAC tapes

1. Health facility Officer

- Record monthly CMAM program admissions and length of stay for cure only.

# **CHAPTER TWO: OVERVIEW OF MALNUTRITION IN MALAWI**

## **What is malnutrition?**

Malnutrition refers both to under-nutrition and over-nutrition (Blössner & de Onis, 2005), but in this guide we will use the term to refer solely to a deficiency of nutrition. It is defined as a deficiency, excess or imbalance in a person’s intake of energy and/ or nutrients (WHO, 2020)

## What causes malnutrition?

Malnutrition is caused by diseases and/or inadequate diet from the six food groups which includes

- Staples (thick porridge with g/nut powder)
- Vegetables (back jack, pumpkin leaves, carrot)
- Fruits (guavas, mangoes, Tangerines, paw paws)
- Animal products (beef, mice, birds, milk, eggs)
- Legumes and nuts (Beans, peas)
- Fats and oils (g/nut powder, avocado pear)

People fail to eat adequately because of famine due to inadequate rains, floods and poverty which makes people to reap few harvests or buy food to satisfy their demand. On the other hand, unhygienic conditions are a cause of diseases which include diarrhea, Malaria and respiratory infections. These diseases makes people to fail to utilize food taken properly hence causing undernutrition (Bizouerne, 2012).

## What are the types of undernutrition?

The following are the types of undernutrition:

1. Acute malnutrition (wasting and nutritional edema)
2. Stunting
3. Underweight (combined measurements of wasting and stunting)
4. Micronutrient deficiencies (hidden hunger)

# CHAPTER THREE: ACUTE MALNUTRITION

## What is acute malnutrition?

Acute malnutrition is a composite term referring to nutritional oedema (kwashiorkor) and wasting (marasmus) and is caused by a decrease in food consumption and/or illness (Bhutta, et al. 2017). Acute malnutrition increases the likelihood of the children to die as it is indicated that children with acute malnutrition are three to nine times more likely to die than well-nourished children (FANTA, 2018).

## What are the clinical forms of acute malnutrition?

According to FANTA (2018), there are three clinical forms of acute malnutrition

- Marasmus- Severe weight loss or wasting
- Kwashiorkor- bloated appearance due to water retention (nutritional oedema)
- Marasmic-kwashiorkor- a combination of both wasting and nutritional oedema

1. ****Wasting

**Supplementary Figure 1:** Wasting (Marasmus) (Alliance for International Medical Action (2016)

## Effects of wasting

- Easily irritated
- Feels tired and hungry
- High risk of deaths
- Impaired cognitive development
- Risks of chronic diseases later in life

1. Nutritional oedema


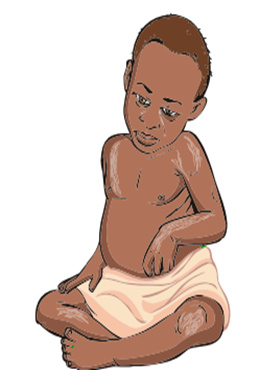


**Supplementary Figure 2:** Oedema (Kwashiorkor) Alliance for International Medical Action (2016)

## Effects of nutritional edema (kwashiorkor)

- Bilateral Pitting oedema on feet, legs, and/or face
- Cracked skin
- Appears sick, sad and does not move much
- Discoloured and brittle hair
- Looks tired
- Loses appetite
- Cries a lot

1. Marasmic-Kwashiorkor

A child with Marasmic-Kwashiorkor shows the following signs and symptoms

- Good appetite
- Old face
- Easily irritated
- Looks tired
- Bilateral pitting oedema on feet, legs/hands and face

## Screening for acute malnutrition

There are three ways of identifying acute malnutrition in 6-59 month children. These are

1. Weight-for-height index- it measures body mass in relation to body height or length.
2. Mid-upper arm circumference (MUAC).
3. Bilateral pitting edema

**Note**

*SCRECA aims at intensifying the use of MUAC and bilateral pitting edema*.

## What is MUAC?

According to Gibson (2005), mid-upper arm circumference (MUAC) is a measurement of the circumference of the upper arm at the midpoint between the olecranon and acromion processes.

## What is the principle behind MUAC?

Since the arm contains both subcutaneous fat and muscle, changes in MUAC can reflect a change in muscle mass, a change in subcutaneous fat, or both. In other words, under conditions of reduced food intake, lower levels of subcutaneous fat and muscle mass in human arms tend to correspond to a decrease in MUAC (Qureshi, Qureshi, Syedd, & Kokku, 2014).

## What do the colour codes on a MUAC tape mean?


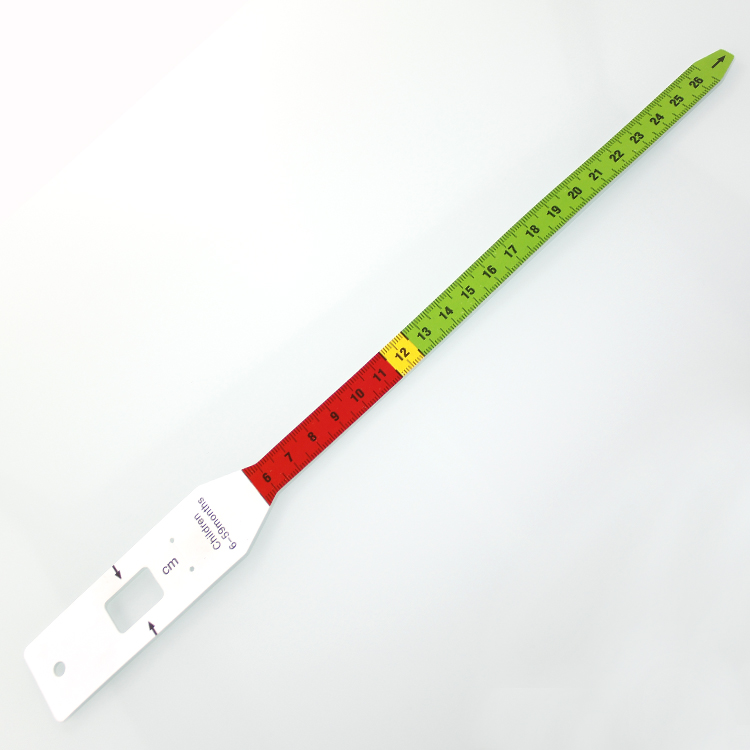


**Supplementary Figure 3:** Alliance for International Medical Action (2016)

## How can one interpret the colour codes on the MUAC tape?


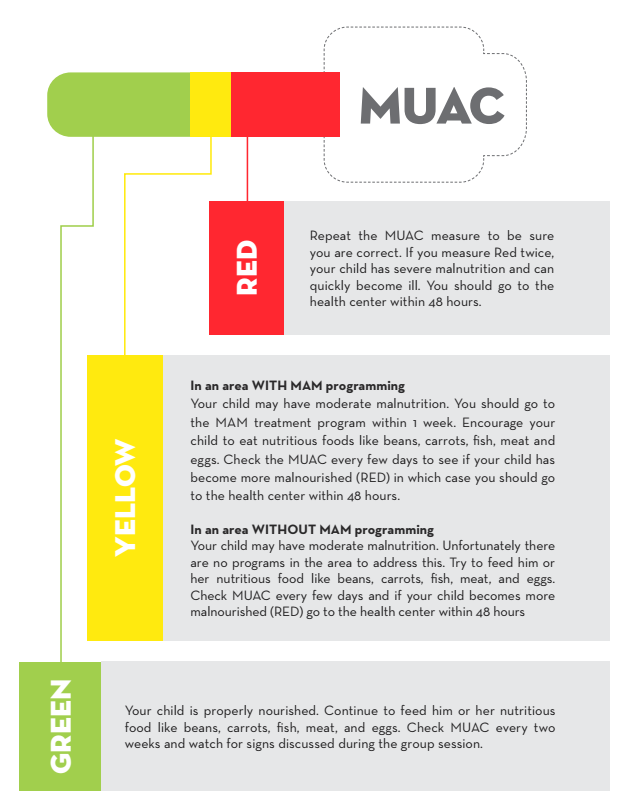


**Supplementary Figure 4:** Alliance for International Medical Action (2016)

## Advantages of using MUAC

Caregivers can detect early signs of malnutrition and seek treatment by regularly checking their child’s MUAC and checking for nutritional edema. Early detection can reduce the risk of death for a child and reduce the risk of a child needing to be hospitalized for an extended period (ALIMA, 2016)

## **How does one take a MUAC measurement?**

*Materials*

A MUAC tape

A string

A pen

**Procedure**
NB: Always remember that MUAC measurement is taken on the left arm.

1. Measure the length of the child’s upper arm, between the bone at the top of the shoulder and the tip of the elbow (the child’s arm should be bent to easily locate the tip).


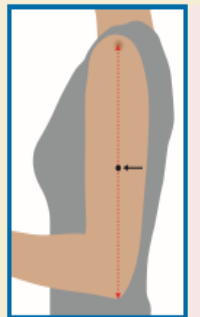


**Supplementary Figure 5:** Community nutrition and dietetics (2017)

1. Find the midpoint of the upper arm and mark it with a pen. It is easier to use a string instead of the MUAC tape to find the midpoint.
2. The child’s arm should then be relaxed, falling alongside his or her body


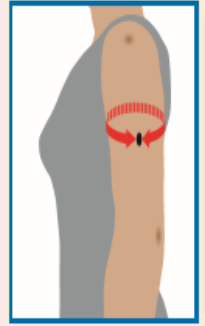


**Supplementary Figure 6:** Community nutrition and dietetics (2017)

1. Wrap the MUAC tape around the child’s arm, so that all of it is in contact with the child’s skin.


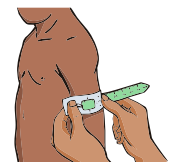


**Supplementary Figure 7: Alliance for International Medical Action (2016)**

1. Feed the end of the tape through the first opening and then through the second opening. It should be neither too tight nor too loose. The measurement is read from the window where the arrows point inward.


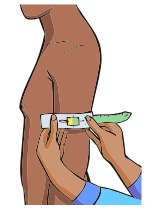


**Supplementary Figure 8: Alliance for International Medical Action (2016)**

1. Record the MUAC reading with a precision of 0.1cm

When should caregivers check MUAC and oedema in their children?

Caregivers should check the child’s MUAC and oedema every two weeks or whenever they
feel it is necessary. It is important to seek treatment at the earliest signs of malnutrition to
reduce the risk of the child dying or needing to be hospitalized.

## How can caregivers ensure safety of the MUAC tape?

- MUAC tapes should be kept in a safe place in the house.
- Do not bend the tape, for example, a MUAC tape can be hung as follows


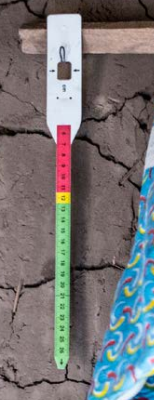


**Supplementary Figure 9: Alliance for International Medical Action (2016)**

**How to check for Nutritional Oedema**

Kwashiorkor, which is also known as oedematous malnutrition or nutritional oedema or bilateral pitting oedema, is an abnormal infiltration and excess accumulation of serous fluid in connective tissue or in a serous cavity (CMAM, 2008). It is a sign of severe malnutrition and it is defined by bilateral pitting oedema of the feet verified when the thumb pressure applied on top of both feet for three seconds leaves a pit in the foot when the thumb is lifted. There are three categories of bilateral pitting oedema and these are mild, moderate and severe. Mild bilateral pitting oedema manifests on both feet and sometimes ankles and it this called grade +. Moderate bilateral pitting manifests on both feet, lower legs, hands or lower arms and this is called grade ++. The severe type is the generalized bilateral pitting oedema including feet, legs, hands, arms and face and it is called grade +++ (ibid). Basically, kwashirkor is caused by shortage of proteins in the body but its pathophysiology is not well understood(Lenters, Wazny and Bhutta, 2016)

**
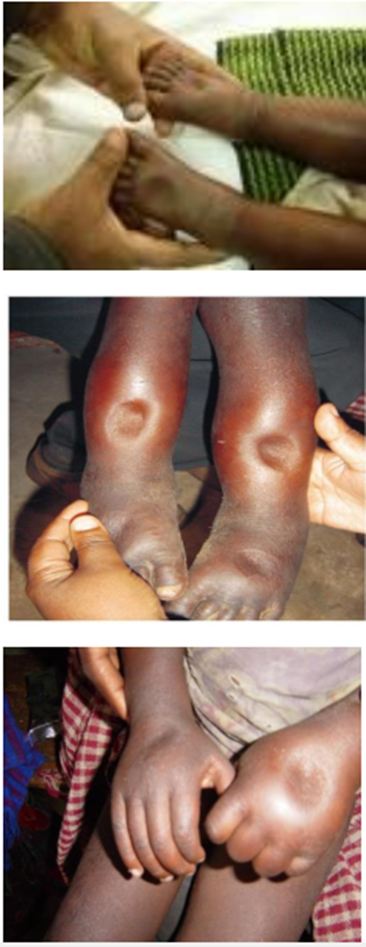
**

**Supplementary Figure 10:** Malawi CMAM Guidelines (2016)

**
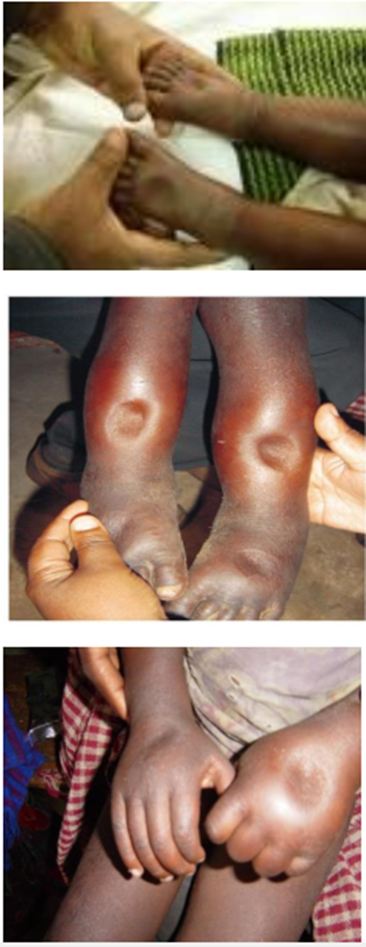
**

**Supplementary Figure 11:** Malawi CMAM Guidelines (2016)

**
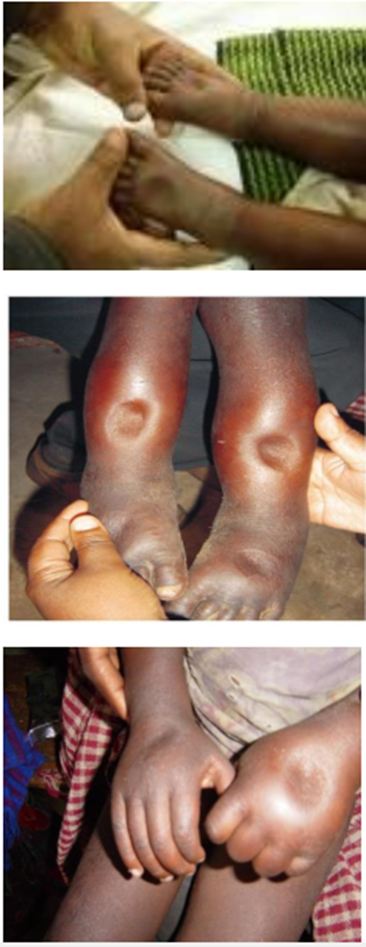
**

**Supplementary Figure 12:** Malawi CMAM Guidelines (2016)

# CHAPTER FOUR: COMMUNITY-BASED MANAGEMENT OF ACUTE MALNUTRITION (CMAM)

## What are the components of CMAM?

1. Community outreach

Improves understanding and stimulates community engagement and participation in malnutrition prevention, identification and treatment.

1. Supplementary feeding programme

Treats MAM in children 6 months to 15 years and pregnant or lactating women with dry, take-home food rations. Children attend an SFP site every two weeks for medical check-up and re-supply of food rations.

1. Outpatient therapeutic programme

Treats MAM in children 6 months to 15 years who present with appetite and without medical complications. Routine medications and Ready-to-Use Therapeutic Food (RUTF) are given to such children as outpatients. Children attend an OTP site weekly for medical check-ups and re-supply of RUTF.

1. Inpatient care management at Nutrition Rehabilitation Unit (NRU)

Provides inpatient care management for children 0 to 15 years with SAM who present with poor appetite and/or medical complications until their condition has stabilized and they are able to continue with SAM treatment in the OTP.

## How does SCRECA fit in CMAM program?

1. Maximum access and coverage

SCRECA aims to increase coverage of acute malnutrition screening and referral through training caregivers. It will introduce and monitor use of MUAC tapes by households with 6-59 month children, and each household with such a child will receive MUAC tapes which will regularly be used to assess the nutritional status of children on an on-going basis to catch as many active cases as possible.

1. Timeliness

SCRECA aims at achieving timeliness by empowering caregivers to screen their children on regular basis to actively and timely identify malnutrition cases which will stimulate early referral and timely treatment. Decentralized services allow for early referral because caregivers can refer to health facilities with outpatient care close to home.

1. Care as long as it is needed

SCRECA intends to replace MUAC tapes that are lost or destroyed to caregivers to enable them demand service when acute malnutrition is detected.

# REFERENCES

Ale, F., Phelan, K. P., Isa, H., Defourny, I., Duc, G., Harczi, G., & Issaley, K. (2016). Mothers screening for malnutrtion by mid-upper arm circumference is inferior to community health workers: results from a large-scale pragmatic trial in rural Niger. *Archives of Public Health, 74*(38). doi:10.1186/s13690-016-0149-5

ALIMA. (2016). *Guidelines for Training Trainers.* France.

Bhadoria, A., Kapil, U., Pandey, R., Pant, B., & Mohan, A. (2017). Prevalence of Severe Acute Malnutrition and Associated Sociodemographic Factors among Children aged 6 Months to 5 Years in Rural Population of Northern India: A Population-Based Survey. *Journal of Family Medicine and Primary Care*. doi:10.4103/jfmpc-421-16

Bizouerne, C. (2012). *Conceptual Models of Child Malnutrition: The ACF Approach in Mental Health and Care Practices.* France: ACF International.

Black, R., Allen, L., Bhutta, Z., Caufield, L., de Onis, M., Ezzati, M., . . . Rivera, J. (2008, January 17). Maternal and Child Undernutrition: Global and Regional Exposures and Health Consequences. *Lancet, 371*, 243-260. doi:10.1016/s0140-673(07)61690-0

Blackwell, N., Myatt, M., Allafort-Duverger, T., Balogoun, A., Ibrahim, A., & Briend, A. (2015). Mother Understand And Can do it (MUAC): a comparison of mothers and community health workers determining mid-upper arm circumference in 103 children aged from 6 months to five years. *Archives of Public Health, 76*(26). doi:10.1186/s13690-015-0074-z

Blössner, M., & de Onis, M. (2005). *Malnutrition: Quantifying the Health Impact at National and Local Levels.* Geneva: World Health Organization.

De Onis, M., Yip, R., & Mei, Z. (1997). The Development of MUAC-for-Age Reference Data Recommended by a WHO Epert Committe. *The Bulletin of World Health Organization, 75*(1), 11-18.

Dunlop, K., Ercolano, F., Giordano, N., Gruening, E., Hunter, J., Lort-Phillips, H., . . . Woolfenden, J. (2019). *Global Performance Report 2018.* UK: Action Against Hunger.

Edriss, A. (2013). *Smart research methods for economics, Business, Health and development.* Lilongwe: Internation i-publishers.

GoM. (2015). *The Cost of Hunger in Malawi: Social and Economic Impacts of Child Undernutrition in Malawi, Implications on National Development and Vision 2020.* COHA Project.

GoM. (2015). *The Cost of Hunger in Malawi; The Social and Economic impact of Child Undernutrition in Malawi.* Lilongwe : Ministry of Finance, Economic Planning and Development .

GoM. (2015-2016). *Malawi Drought 2015-2016: Post-Disaster Needs Assessment.*

GoM. (2016). *Malawi Drought 2015-2016: Post-Disaster Needs Assessment.*

Laillou, A., Prak, S., de Groot, R., Whitney, S., Conkle, J., Horton, L., . . . Wieringa, F. (2014, July). Optimal Screening of Children with Acute Malnutrition Requires a Change in Current WHO Guidelines as MUAC and WHZ Identify Different Patient Groups. *PLOS ONE, 9*(7). doi:10.1371/journal.pone0101159

Myatt, M., Guevarra, E., Fieschi, L., Norris, A., Guerrero, S., Schofield, L., . . . Sadler, K. (2012). *Semi-Quantitative Evaluation of Access and Coverage (SQUEAC)/ Simplified Lot Quality Assurance Sampling Evaluation of Access and Coverage (SLEAC) Techinical Reference.* Washington, DC: FANTA.

NSO. (2019). *2018 Malawi and Housing Census Report.* Zomba.

Ramachandran, P., & Gopalan, H. (2009). Undernutrtion and risk of infections in preschool children. *Indian J Med Res, 130*, 579-583.

UNICEF. (2019). *Malawi Floods Situation Report.* UNICEF.

UNICEF. (2019). *Malawi Floods Situation Report.*

UNICEF. (2019). *The State of World's Children 2019: Children, Food and Nutrition.* New York: UNICEF.

WHO. (2019). *Strategic Plan to Reduce the Double Burden of Malnutrition in the African Region: 2019-2025.* Brazzaville, Republic of Congo: WHO/AFR/RC69/7.

WHO. (2020, January 09). *Malnutrition*. Retrieved from World Health Organization: https//www.who.int/news-room/fact-sheets/detail/malnutrition

**SCRECA PROJECT**

TRAINING HANDBOOK FOR CAREGIVERS

**TRAINING GUIDE**

**Supplementary Table 1: Introduction**

| **PART 1** | | |
| --- | --- | --- |
| **FACILITATOR’S ACTIVITIES** | **CAREGIVERS’ ACTIVITIES** | **MATERIALS** |
| **Introduction (5 minutes)**   - Welcoming caregivers and directing them to take their seats - Introducing himself/herself to caregivers and requesting caregivers to do the same - Ice breaker - Ask any caregiver to lead the group in a song | - Following the facilitator’s instructions - Introducing themselves - Singing |  |

**Supplementary Table 2: Causes and prevention of malnutrition**

| **PART 2** | | |
| --- | --- | --- |
| **FACILITATOR’S ACTIVITIES** | **CAREGIVERS’ ACTIVITIES** | **MATERIALS** |
| **STEP 1 (5 minutes)**  **Method: whole group discussion**   - Explain the aim of the training - To train caregivers how to assess the nutritional status of their children aged 6-59 months using MUAC and oedema - Ask caregivers what malnutrition is - Ask caregivers forms of malnutrition - Show caregivers different forms of undernutrition on the posters - Consolidation | - Listening - Giving the definition of malnutrition - A deficiency, excess or imbalance in a person’s intake of energy and/ or nutrients (WHO, 2020) - Mentioning forms of malnutrition - Overnutrition - Undernutrition- acute malnutrition (kwashiorkor, marasmus), stunting, underweight and micronutrient deficiencies | Charts/ posters showing different forms of malnutrition |
| **STEP 2 (5 minutes)**  **Method: whole group discussion**   - Ask caregivers what causes undernutrition in their community - Explain the dangers of undernutrition to children - Death - Sickness - Poor performance in school - Ice breaker- ask caregivers for an ice breaker - consolidation | - Mentioning the causes - Failing to practice exclusive breastfeeding - Poor hygiene which causes infections - Food insecurity which prevents them to give enough food to their children - Lack of knowledge on cooking nutritious food |  |
| **STEP 3 (5 minutes)**  **Method: lecture**  Explain to caregivers the foods which they need to give their children and the quantities   - Beans, porridge with g/nut flour, nuts, carrots, eggs, milk, vegetables and fruits, local foods such as ziwala, mbewa. - Let caregivers add on the list based on foods they have in their homes | - Listening and giving their views based on experience | Poster with various foods if available |

**Supplementary Table 3: Categories of malnutrition**

| **PART 3** | | |
| --- | --- | --- |
| **FACILITATOR’S ACTIVITIES** | **CAREGIVERS’ ACTIVITIES** | **MATERIALS** |
| **STEP 1 (5 minutes)**   - Explain to caregivers the ways of recognizing signs of malnutrition - Low MUAC (thinness) and oedema   **Wasting (Marasmus)**   - Easily irritated - Feels tired - Older looking face - Muscle wasting, skin sticks to the bones - Still has an appetite   - Sometimes has oedema on both feet, legs, and/or face   **Oedema (kwashiorkor)**   - Pitting oedema on feet, legs, and/or face - Cracked skin - Appears sick, sad and does not move much - Discoloured and brittle hair - Looks tired - Loses appetite   - Cries a lot | - Listening and contributing their ideas based on experience | Charts/ posters showing children suffering from marasmus and kwashiorkor |
| **STEP 2 (5 minutes)**   - Explain the aim of Family-MUAC - Empowers mothers/caregivers to track their child’s growth closely - Allows family members to assess the nutritional status of children aged 6-59 months on regular basis - Allows caregivers to monitor the health of the child - Enables caregivers to seek health services on time when they discover that the child is malnourished - Explain the benefits of Family-MUAC - Allows caregivers to detect early signs of malnutrition and reduce the risk of a child needing to be hospitalized for long period of time - Early detection can reduce the risk of death for a child - Easy to use and convenient - Ice breaker | - Listening | Posters showing mothers assessing their children with a MUAC tape, MUAC tape |

**Supplementary Table 4:** Instructions of MUAC assessment by caregivers

| **PART 4** | | |
| --- | --- | --- |
| **FACILITATOR’S ACTIVITIES** | **CAREGIVERS’ ACTIVITIES** | **MATERIALS** |
| **STEP1 (5 minutes)**  **Method: explanation/demonstration**   - Tell caregivers that they are going to learn how to take MUAC measurement and how to check oedema in their children - Tell caregivers to look at the MUAC tape and identify colours on it   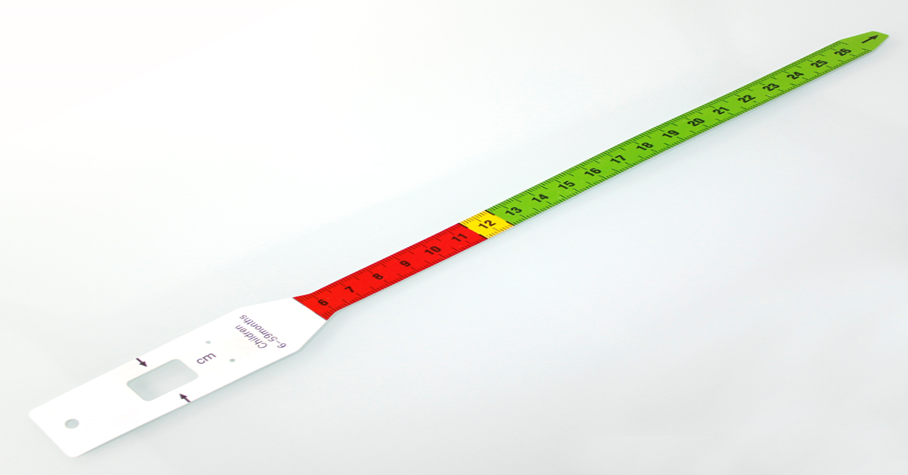  **Supplementary Figure 13:** Modified from Alliance for International Medical Action (2016) | - Following the facilitator’s instructions - Identifying colours on the tape- green, yellow and red | MUAC tape  Oedema mock-up  String |
| **STEP 2 (5 minutes)**  **Method: explanation**   - Tell mothers what they should do when their children are normal or have MAM and SAM   **Normal MUAC (green)**   - Caregivers should continue feeding their children with good nutritious food   **MAM (yellow**)   - Caregivers should take the child to health surveillance assistant within 1 week who will refer the child to health facility for SFP - Give the child nutritious food such as beans, peas, porridge, meat, eggs, fruits and vegetables   **SAM (red)**   - Go to see the HSA who will refer you the health centre. This should be done within 48 hours because the child is at risk of becoming ill - Consolidation | - Listening | MUAC tapes  Posters of kwashiorkor and marasmus |
| **STEP 3 (5 minutes)**  **Method: explanation/demonstration**   - Explain how MUAC tape is used, if possible, demonstrate on a baby doll or a real baby - Always remember that MUAC measurement is taken on the left arm - Measure the length of the child’s upper arm, between the bone at the top of the shoulder and the tip of the elbow (the child’s arm should be bent to easily locate the tip).   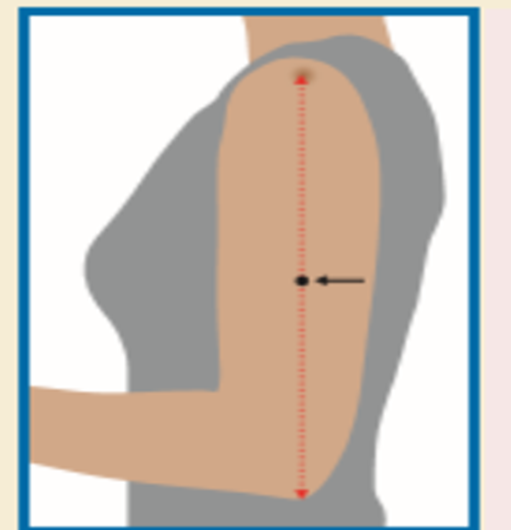  **Supplementary Figure 14:** Community nutrition and dietetics (2017)   - Find the midpoint of the upper arm and mark it with a pen. It is easier to use a string instead of the MUAC tape to find the midpoint. - The child’s arm should then be relaxed, falling alongside his or her body   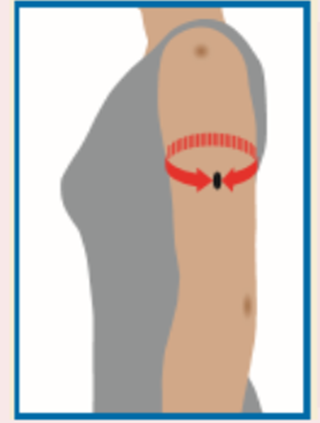  **Supplementary Figure 15:** Community nutrition and dietetics (2017)   - Wrap the MUAC tape around the child’s arm, so that all of it is in contact with the child’s skin.   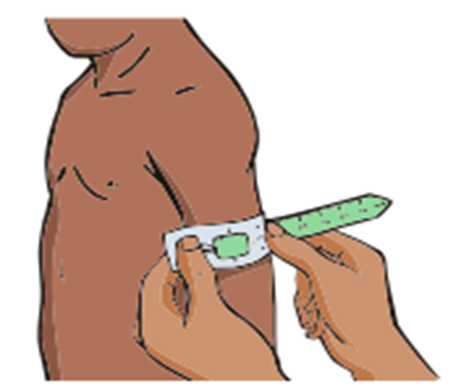  **Supplementary Figure 16:** Alliance for International Medical Action (2016)   - Feed the end of the tape through the first opening and then through the second opening. It should be neither too tight nor too loose. The measurement is read from the window where the arrows point inward.   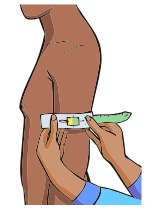  **Supplementary Figure 17:** Alliance for International Medical Action (2016)   - Record the MUAC colour code - Tell caregivers to practice the process and help them where necessary - Go round and record the caregiver’s colour codes - Take each child’s MUAC and record the results - Consolidation | - Listening - Practice taking MUAC measurements on their children and reporting the colour code read to facilitator - Asking for clarification when necessary | Baby doll if available  MUAC tape |
| **STEP 4 (5 minutes)**  **Checking for oedema**   - Tell caregivers that oedema is a sign of severe malnutrition and has three categories - Mild (feet) - Moderate (feet, hands) - Severe (feet, arms and face) - Tell caregivers that oedema is verified when the thumb pressure applied on top of both feet for three seconds leaves a pit in the foot when the thumb is lifted - Demonstrate how to check for oedema on the feet and hands- you can use oedema mock-up - Tell caregivers that they should not check for oedema of the face of the children - Tell caregivers to practice checking for oedema on their children - Check for oedema on each child and record the results - Consolidation | - Listening - Practicing checking for oedema | Oedema mock-up |
| **STEP 5 (5 minutes)**   - Tell caregivers when to check their children’s MUAC and oedema - Every two weeks or whenever they feel it is necessary. - It is important to seek treatment at the earliest signs of malnutrition to decrease the risk of your child dying or needing to be hospitalized - Ask caregivers how they will keep their MUAC tape to avoid loss or destroying - Show them how others keep it   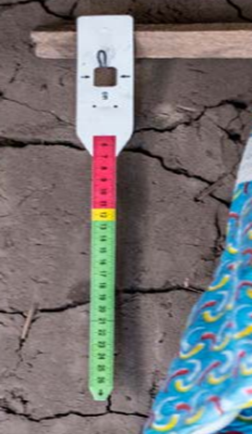  **Supplementary Figure 18:** Alliance for International Medical Action (2016) | - Listening - Giving views on how they intend to keep the MUAC tape |  |
| **STEP 6 (5 minutes)**  **CONCLUSION**   - Ask caregivers what they have learned | - Giving points grasped - Definition of malnutrition and its causes - Advantage of family MUAC - How take MUAC measurement and checking for oedema |  |

# REFERENCES

Ale, F., Phelan, K. P., Isa, H., Defourny, I., Duc, G., Harczi, G., & Issaley, K. (2016). Mothers screening for malnutrtion by mid-upper arm circumference is inferior to community health workers: results from a large-scale pragmatic trial in rural Niger. *Archives of Public Health, 74*(38). doi:10.1186/s13690-016-0149-5

Alliance for International Medical Action (2016). *Guidelines for Training Trainers.* France.

Bhadoria, A., Kapil, U., Pandey, R., Pant, B., & Mohan, A. (2017). Prevalence of Severe Acute Malnutrition and Associated Sociodemographic Factors among Children aged 6 Months to 5 Years in Rural Population of Northern India: A Population-Based Survey. *Journal of Family Medicine and Primary Care*. doi:10.4103/jfmpc-421-16

Bizouerne, C. (2012). *Conceptual Models of Child Malnutrition: The ACF Approach in Mental Health and Care Practices.* France: ACF International.

Black, R., Allen, L., Bhutta, Z., Caufield, L., de Onis, M., Ezzati, M., . . . Rivera, J. (2008, January 17). Maternal and Child Undernutrition: Global and Regional Exposures and Health Consequences. *Lancet, 371*, 243-260. doi:10.1016/s0140-673(07)61690-0

Blackwell, N., Myatt, M., Allafort-Duverger, T., Balogoun, A., Ibrahim, A., & Briend, A. (2015). Mother Understand And Can do it (MUAC): a comparison of mothers and community health workers determining mid-upper arm circumference in 103 children aged from 6 months to five years. *Archives of Public Health, 76*(26). doi:10.1186/s13690-015-0074-z

Blössner, M., & de Onis, M. (2005). *Malnutrition: Quantifying the Health Impact at National and Local Levels.* Geneva: World Health Organization.

De Onis, M., Yip, R., & Mei, Z. (1997). The Development of MUAC-for-Age Reference Data Recommended by a WHO Epert Committe. *The Bulletin of World Health Organization, 75*(1), 11-18.

Dunlop, K., Ercolano, F., Giordano, N., Gruening, E., Hunter, J., Lort-Phillips, H., . . . Woolfenden, J. (2019). *Global Performance Report 2018.* UK: Action Against Hunger.

Edriss, A. (2013). *Smart research methods for economics, Business, Health and development.* Lilongwe: Internation i-publishers.

GoM. (2015). *The Cost of Hunger in Malawi: Social and Economic Impacts of Child Undernutrition in Malawi, Implications on National Development and Vision 2020.* COHA Project.

GoM. (2015). *The Cost of Hunger in Malawi; The Social and Economic impact of Child Undernutrition in Malawi.* Lilongwe : Ministry of Finance, Economic Planning and Development .

GoM. (2015-2016). *Malawi Drought 2015-2016: Post-Disaster Needs Assessment.*

GoM. (2016). *Malawi Drought 2015-2016: Post-Disaster Needs Assessment.*

Laillou, A., Prak, S., de Groot, R., Whitney, S., Conkle, J., Horton, L., . . . Wieringa, F. (2014, July). Optimal Screening of Children with Acute Malnutrition Requires a Change in Current WHO Guidelines as MUAC and WHZ Identify Different Patient Groups. *PLOS ONE, 9*(7). doi:10.1371/journal.pone0101159

Myatt, M., Guevarra, E., Fieschi, L., Norris, A., Guerrero, S., Schofield, L., . . . Sadler, K. (2012). *Semi-Quantitative Evaluation of Access and Coverage (SQUEAC)/ Simplified Lot Quality Assurance Sampling Evaluation of Access and Coverage (SLEAC) Techinical Reference.* Washington, DC: FANTA.

NSO. (2019). *2018 Malawi and Housing Census Report.* Zomba.

Ramachandran, P., & Gopalan, H. (2009). Undernutrtion and risk of infections in preschool children. *Indian J Med Res, 130*, 579-583.

UNICEF. (2019). *Malawi Floods Situation Report.* UNICEF.

UNICEF. (2019). *Malawi Floods Situation Report.*

UNICEF. (2019). *The State of World's Children 2019: Children, Food and Nutrition.* New York: UNICEF.

WHO. (2019). *Strategic Plan to Reduce the Double Burden of Malnutrition in the African Region: 2019-2025.* Brazzaville, Republic of Congo: WHO/AFR/RC69/7.

WHO. (2020, January 09). *Malnutrition*. Retrieved from World Health Organization: https//www.who.int/news-room/fact-sheets/detail/malnutrition
